# Causes of death and associated factors over a decade of follow-up in a cohort of people living with HIV in rural Tanzania

**DOI:** 10.1186/s12879-021-06962-3

**Published:** 2022-01-06

**Authors:** Getrud Joseph Mollel, Lilian Moshi, Hoda Hazem, Anna Eichenberger, Olivia Kitau, Herry Mapesi, Tracy R. Glass, Daniel Henry Paris, Maja Weisser, Fiona Vanobberghen

**Affiliations:** 1 Ifakara Health Institute, Ifakara, Tanzania; 2 St. Francis Referral Hospital, Ifakara, Tanzania; 3 Swiss Tropical and Public Health Institute, Basel, Switzerland; 4 University of Basel, Basel, Switzerland; 5 Department of Infectious Diseases, Bern University Hospital, University of Bern, Bern, Switzerland; 6 Division of Infectious Diseases and Hospital Epidemiology, University Hospital Basel, Basel, Switzerland

**Keywords:** HIV infections, Cause of death, Tanzania, Mortality, Cohort, Proportional hazards models

## Abstract

**Background:**

Nearly half of HIV-related deaths occur in East and Southern Africa, yet data on causes of death (COD) are scarce. We determined COD and associated factors among people living with HIV (PLHIV) in rural Tanzania.

**Methods:**

PLHIV attending the Chronic Diseases Clinic of Ifakara, Morogoro are invited to enrol in the Kilombero and Ulanga Antiretroviral Cohort (KIULARCO). Among adults (≥ 15 years) enrolled in 2005–2018, with follow-up through April 2019, we classified COD in comprehensive classes and as HIV- or non-HIV-related. In the subset of participants enrolled in 2013–2018 (when data were more complete), we assessed cause-specific mortality using cumulative incidences, and associated factors using proportional hazards models.

**Results:**

Among 9871 adults (65% female, 26% CD4 count < 100 cells/mm^3^), 926 (9%) died, among whom COD were available for 474 (51%), with missing COD mainly in earlier years. The most common COD were tuberculosis (N = 127, 27%), non-AIDS-related infections (N = 72, 15%), and other AIDS-related infections (N = 59, 12%). Cardiovascular and renal deaths emerged as important COD in later calendar years, with 27% of deaths in 2018 attributable to cardiovascular causes. Most deaths (51%) occurred within the first six months following enrolment. Among 3956 participants enrolled in 2013–2018 (N = 203 deaths, 200 with COD ascertained), tuberculosis persisted as the most common COD (25%), but substantial proportions of deaths from six months after enrolment onwards were attributable to renal (14%), non-AIDS-related infections (13%), other AIDS-related infections (10%) and cardiovascular (10%) causes. Factors associated with higher HIV-related mortality were sex, younger age, living in Ifakara town, HIV status disclosure, hospitalisation, not being underweight, lower CD4 count, advanced WHO stage, and gaps in care. Factors associated with higher non-HIV-related mortality included not having an HIV-positive partner, lower CD4 count, advanced WHO stage, and gaps in care.

**Conclusion:**

Incidence of HIV-related mortality was higher than that of non-HIV-related mortality, even in more recent years, likely due to late presentation. Tuberculosis was the leading specific COD identified, particularly soon after enrolment, while in later calendar years cardiovascular and renal causes emerged as important, emphasising the need for improved screening and management.

**Supplementary Information:**

The online version contains supplementary material available at 10.1186/s12879-021-06962-3.

## Introduction

Globally, 75 million people have been infected with HIV since its emergence and 32.7 million have died, as of 2019 [1, 2]. Sub-Saharan Africa (SSA) harbours over two-thirds of the global population of people living with HIV (PLHIV), representing the most affected region [2], despite being home to only 14% of the world’s total population. AIDS-related mortality has declined by 39% since 2010, yet in 2019 alone it is estimated that there were 690,000 AIDS-related deaths, with 300,000 of them in the East and Southern Africa regions [2].

The life expectancy of PLHIV is improving due to the scale-up of antiretroviral therapy (ART) [3, 4]. While the risks of dying from HIV-related causes are decreasing in most settings, non-HIV-related conditions become more important [5, 6]. A study conducted among PLHIV on ART attending an urban clinic in Uganda in 2002–2012 observed a shift over time in the predominant causes of death from communicable and AIDS-defining malignancies to non-communicable conditions [7]. A further study in Uganda found that 30% of deaths were attributable to non-communicable diseases including arterial hypertension and diabetes mellitus, while tuberculosis and cryptococcosis were the main causes of HIV-related mortality [8]. A study conducted in Barcelona in 2001–2013 found similar rates of HIV-related and non-HIV-related causes of deaths (COD) [9]. A higher incidence of non-AIDS-defining cancers was observed among PLHIV in Canada, compared to people living without HIV [10].

With HIV treatment improving over time, it is necessary to understand the COD among PLHIV in order to optimise clinical care for HIV/AIDS and associated comorbidities. Appropriate screening for and treatment of the most important comorbidities in an aging population of persons on ART prevents morbidity and mortality [11]. To our knowledge, there has been no comprehensive assessment of COD among PLHIV in Tanzania, and data from Eastern and Southern Africa region remain scarce. We evaluated COD among PLHIV enrolled in the Kilombero and Ulanga Antiretroviral Cohort (KIULARCO) in rural Tanzania, and factors associated with cause-specific mortality.

## Methods

### Study setting and population

The Chronic Diseases Clinic of Ifakara (CDCI) was established in 2005 to support the National AIDS Control Programme of Tanzania. The CDCI is accessible to over one million people residing in the Kilombero and Ulanga districts of the Morogoro region in south-western Tanzania. PLHIV attending the CDCI are invited to enrol into KIULARCO, which has been described in detail elsewhere [12, 13]. In brief, data including demographic characteristics, ART use and treatment outcomes are captured prospectively since 2005, and more comprehensively after the introduction of electronic medical records (https://openmrs.org/) at the end of 2012. This study included adults (aged ≥ 15 years) enrolled during 2005–2018, with follow-up visits through April 2019. Analyses of cause-specific rates and associated factors were restricted to participants enrolled from 2013 onwards due to most COD being unknown before the introduction of electronic medical records at the end of 2012.

### Measurements and definitions

Time was measured from enrolment to death, with follow-up censored at the earliest of database censoring in April 2019, date of lost to follow-up (LTFU; defined as > 60 days late for a scheduled visit, with visits scheduled every 6 months before starting ART and every 3 months once ART was initiated), or date of transfer to another clinic. The underlying COD was defined as the condition that caused or initiated the process of death, as assigned by the attending clinician according to the presenting signs and symptoms and laboratory results for hospitalised patients, or through a combination of information reported by relatives and village health workers and medical records for participants who died at home. COD were reported in the electronic medical records according to the international cause of death codes, tenth version (ICD-10) [14]. COD were categorised into broad categories: HIV-related, non-HIV-related, or unknown cause of death; with sub-classification into AIDS, cardiovascular, AIDS-related infections (other than tuberculosis), tuberculosis, non-AIDS-related infections, AIDS-related malignancies, other malignancies, hepatic causes, renal causes, and others (Additional file 1: Table S1). COD classification was done by two clinicians at the CDCI (GJM and LM), independently reviewed by a third clinician (MW), and subsequently re-reviewed in discussion with two independent clinicians at the CDCI. In general, WHO stage III/IV defining conditions were considered HIV-related.

Baseline variables were gender, age, education, occupation, marital status, distance of residence from the clinic, partner HIV status, HIV status disclosure, smoking, alcohol use, referral as hospital in-patient, pregnancy, body mass index [BMI, categorised as underweight (< 18.5 kg/m^2^), normal (18.5– < 25 kg/m^2^) or overweight (≥ 25 kg/m^2^)] [15], CD4 cell count, WHO stage, tuberculosis (defined as tuberculosis if, within 3 months from enrolment: detection of acid-fast bacilli or positive Xpert MTB/RIF assay (Cepheid, Sunnyvale, CA, USA) from a sputum or an extra-pulmonary sample, or prescription of anti-tuberculosis medication with physician diagnosis indicated by ICD-10 code or clinical signs suggestive of tuberculosis; defined as tuberculosis unlikely if no prescription of anti-tuberculosis drugs and no physician diagnosis; otherwise indeterminate tuberculosis status and treated as missing data), arterial hypertension (if, within 13 weeks from enrolment: systolic blood pressure ≥ 140 mmHg or diastolic blood pressure ≥ 90 mmHg on two consecutive occasions; physician diagnosis indicated by ICD-10 code; or prescription of anti-hypertensive medication) and enrolment year. Participants were considered to have been started on ART at baseline if they initiated before or within 30 days of enrolment. Time-dependent variables were time since ART initiation (updated at each follow-up visit) and previous number of previous gaps in care (periods of being LTFU before returning to care).

### Statistical methods

Descriptive statistics were used to summarise baseline characteristics and outcomes. We estimated HIV-related and non-HIV-related cause-specific mortality rates using cumulative incidences [16, 17]. We assessed factors associated with cause-specific mortality using Cox proportional hazard models [16]. Participants who were LTFU, transferred out to another clinic, or were still under active follow-up at the time of database closure were censored at the time of LTFU, transfer out, or database closure, respectively. Further, in the models with the outcome of HIV-related deaths, participants who died from non-HIV-related causes were censored at the time of their death; and vice versa for the outcome of non-HIV-related deaths. Participants could re-enter the risk set if they returned to care after being LTFU or having transferred out to another clinic. We used multiple imputation with chained equations to address missing baseline covariates, assuming that data were missing at random [18]. In the imputations, we used multinomial regression for distance, partner status and WHO stage; truncated regression for inverse-square-root-transformed BMI and square-root-transformed CD4 count; and logistic regression for disclosure, smoking and tuberculosis. In addition to the baseline covariates, we included in the imputations time spent on ART over all follow-up, an indicator for outcome (died due to HIV-related, non-HIV-related or unknown causes, or censored), and the Nelson-Aalen estimator of the baseline cumulative hazard [18, 19]. We used 10 imputations, based on the rule of thumb that the number of imputations should be equal to or greater than the fraction of missing information [20]. Analyses were conducted in Stata version 15 [21].

## Results

Among 9781 adults, 6315 (65% of those with non-missing information) were female, 3886 (40%) were aged < 35 years, 5787 (93%) had at most primary education, 5269 (85%) were farmers, and 5309 (55%) were married or cohabiting (Table 1). At baseline, among those with data, 3608 (39%) participants lived within 1 km of the clinic, 1376 (15%) had an HIV-positive partner, 5634 (70%) had disclosed their HIV status, 214 (2%) were current smokers and 2099 (24%) reported alcohol consumption (higher in earlier years when participants were asked to respond never or yes, whereas from 2013 onwards, the responses captured were regular/current consumption or not). A minority of participants (N = 774, 8%) were referred from in-patient care, 1895 (24%) were underweight and 1198 (15%) were overweight, and 390 (6% of women) were pregnant. CD4 count was < 100 cells/mm^3^ in 1576 (26%) participants, 3715 (40%) participants were WHO stage III/IV, 539 (6%) had tuberculosis co-infection, and 631 (6%) had arterial hypertension. Half of participants (N = 5041, 52%) initiated ART within 30 days of enrolment.

**Table 1:** Baseline characteristics among adults enrolled in KIULARCO in 2005–2018

Characteristic	Censored†	Died from non-HIV-related cause	Died from HIV-related cause	Died from unknown cause	Total
Total	8855 (91%)	166 (2%)	245 (3%)	515 (5%)	9781 (100%)
Sex					
Male	3049 (89%)	73 (2%)	127 (4%)	191 (6%)	3440 (100%)
Female	5782 (92%)	93 (1%)	118 (2%)	322 (5%)	6315 (100%)
Missing	24 (92%)	0 (0%)	0 (0%)	2 (8%)	26 (100%)
Age, years					
15–24	729 (92%)	7 (1%)	20 (3%)	36 (5%)	792 (100%)
25–34	2817 (91%)	54 (2%)	69 (2%)	154 (5%)	3094 (100%)
35–44	3045 (91%)	53 (2%)	79 (2%)	185 (6%)	3362 (100%)
≥ 45	2264 (89%)	52 (2%)	77 (3%)	140 (6%)	2533 (100%)
Highest education level					
None/primary	5527 (96%)	96 (2%)	115 (2%)	49 (1%)	5787 (100%)
Beyond primary	420 (97%)	4 (1%)	11 (3%)	0 (0%)	435 (100%)
Missing	2908 (82%)	66 (2%)	119 (3%)	466 (13%)	3559 (100%)
Occupation					
Farmer	5031 (95%)	88 (2%)	108 (2%)	42 (1%)	5269 (100%)
Not farmer	916 (96%)	12 (1%)	18 (2%)	7 (1%)	953 (100%)
Missing	2908 (82%)	66 (2%)	119 (3%)	466 (13%)	3559 (100%)
Marital status					
Married/cohabiting	4866 (92%)	94 (2%)	113 (2%)	236 (4%)	5309 (100%)
Never married	1178 (87%)	22 (2%)	44 (3%)	115 (8%)	1359 (100%)
Separated/divorced/ widowed/others	2686 (92%)	43 (2%)	76 (3%)	129 (4%)	2934 (100%)
Missing	125 (70%)	7 (4%)	12 (7%)	35 (20%)	179 (100%)
Distance of residence from clinic, km					
≤ 1 (i.e., resident in Ifakara town)	3179 (88%)	82 (2%)	115 (3%)	232 (6%)	3608 (100%)
2– < 50	2600 (95%)	42 (2%)	47 (2%)	51 (2%)	2740 (100%)
≥ 50	2577 (91%)	30 (1%)	63 (2%)	147 (5%)	2817 (100%)
Missing	499 (81%)	12 (2%)	20 (3%)	85 (14%)	616 (100%)
Partner HIV sero-status					
Positive	1301 (95%)	18 (1%)	20 (1%)	37 (3%)	1376 (100%)
Negative	687 (92%)	16 (2%)	19 (3%)	28 (4%)	750 (100%)
Unknown	4941 (88%)	99 (2%)	150 (3%)	413 (7%)	5603 (100%)
Not applicable	1277 (93%)	27 (2%)	49 (4%)	16 (1%)	1369 (100%)
Missing	649 (95%)	6 (1%)	7 (1%)	21 (3%)	683 (100%)
HIV status disclosure					
Not disclosed	2224 (93%)	32 (1%)	43 (2%)	97 (4%)	2396 (100%)
Disclosed	5160 (92%)	94 (2%)	146 (3%)	234 (4%)	5634 (100%)
Missing	1471 (84%)	40 (2%)	56 (3%)	184 (11%)	1751 (100%)
Smoking					
Never/stopped	7871 (91%)	157 (2%)	222 (3%)	443 (5%)	8693 (100%)
Current	196 (92%)	1 (0%)	6 (3%)	11 (5%)	214 (100%)
Missing	788 (90%)	8 (1%)	17 (2%)	61 (7%)	874 (100%)
Alcohol use^¥^					
No	6097 (91%)	112 (2%)	178 (3%)	290 (4%)	6677 (100%)
Yes	1869 (89%)	41 (2%)	48 (2%)	141 (7%)	2099 (100%)
Missing	889 (88%)	13 (1%)	19 (2%)	84 (8%)	1005 (100%)
Patient referred from in-patient care hospitalisation					
No	7890 (91%)	137 (2%)	181 (2%)	424 (5%)	8632 (100%)
Yes	688 (89%)	21 (3%)	44 (6%)	21 (3%)	774 (100%)
Missing	277 (74%)	8 (2%)	20 (5%)	70 (19%)	375 (100%)
Pregnant*					
No	5409 (91%)	89 (2%)	113 (2%)	314 (5%)	5925 (100%)
Yes	373 (96%)	4 (1%)	5 (1%)	8 (2%)	390 (100%)
BMI, kg/m^2^‡					
Underweight (< 18.5)	1643 (87%)	40 (2%)	58 (3%)	154 (8%)	1895 (100%)
Normal (18.5- < 25)	4416 (92%)	80 (2%)	118 (2%)	170 (4%)	4784 (100%)
Overweight (≥ 25)	1148 (96%)	10 (1%)	12 (1%)	28 (2%)	1198 (100%)
Missing	1275 (84%)	32 (2%)	52 (3%)	155 (10%)	1514 (100%)
CD4 count, cells/mm^3^					
< 100	1347 (85%)	48 (3%)	82 (5%)	99 (6%)	1576 (100%)
100–349	2331 (94%)	35 (1%)	52 (2%)	74 (3%)	2492 (100%)
≥ 350	1888 (96%)	23 (1%)	21 (1%)	42 (2%)	1974 (100%)
Missing	3289 (88%)	60 (2%)	90 (2%)	300 (8%)	3739 (100%)
WHO stage					
I/II	5105 (94%)	73 (1%)	50 (1%)	214 (4%)	5442 (100%)
III	2218 (89%)	48 (2%)	91 (4%)	148 (6%)	2505 (100%)
IV	960 (79%)	38 (3%)	93 (8%)	119 (10%)	1210 (100%)
Missing	572 (92%)	7 (1%)	11 (2%)	34 (5%)	624 (100%)
Tuberculosis					
Unlikely	8205 (91%)	149 (2%)	188 (2%)	504 (6%)	9046 (100%)
Yes	477 (88%)	12 (2%)	44 (8%)	6 (1%)	539 (100%)
Missing	173 (88%)	5 (3%)	13 (7%)	5 (3%)	196 (100%)
Arterial hypertension					
No	8261 (90%)	153 (2%)	232 (3%)	504 (6%)	9150 (100%)
Yes	594 (94%)	13 (2%)	13 (2%)	11 (2%)	631 (100%)
ART status (within 30 days of enrolment)					
Not yet initiated ART	4171 (88%)	85 (2%)	144 (3%)	340 (7%)	4740 (100%)
Initiated ART	4684 (93%)	81 (2%)	101 (2%)	175 (3%)	5041 (100%)

Results are number and row percentage. †Due to administrative censoring at time of database closure, LTFU or transfer out to another clinic (see “Methods”). ^¥^Captured as ever versus never in 2005–2012, and regular/current versus not regular/current from 2013 onwards. *Percentages are of females. ^‡^Pregnant women excluded

**Fig. 1:**
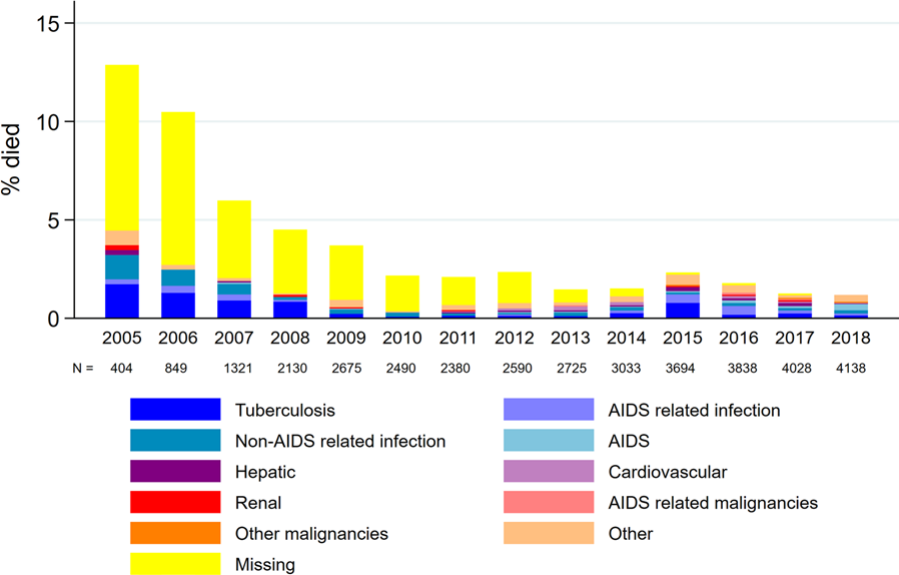
Causes of death among adults enrolled in KIULARCO in 2005–2018, by calendar year. Percentage died calculated as the number of deaths in a given calendar year divided by the number of participants who attended a clinic visit during that year (which is indicated by N below the graph). The category “AIDS-related infections” excludes tuberculosis which is presented separately

The median follow-up time was 2.0 years (interquartile range 0.7–5.7). Overall, 3563 (36%) participants were in active care at database censoring, 1341 (14%) had transferred out to other clinics, 3951 (40%) were LTFU, and 926 (9%) had died. The proportion of participants who died (among those who had follow-up visits in a given year) declined steadily over time, from 13% in 2005 to 1% in 2018 (Fig. 1). Most deaths (N = 470, 51%) occurred within the first 6 months following enrolment.

COD was ascertained for 474 participants (51% of those who died), with missing COD mainly in earlier years (Fig. 1). The most common causes of death were tuberculosis (N = 127, 27% of those with COD), non-AIDS-related infections (N = 72, 15%), and other AIDS-related infections (N = 59, 12%). The most common non-AIDS-related infection COD were malaria (N = 26) and pneumonia (N = 24) while other AIDS-related infection COD (excluding tuberculosis) were mainly cryptococcosis (N = 32) and suspected *Pneumocystis jirovecii* pneumonia (N = 21; Additional file 1: Table S1). Notably, cardiovascular and renal causes emerged as important COD in later calendar years. In 2018, 27% of deaths were attributed to cardiovascular causes compared to ≤ 8% in previous years. In 2015–2018, 6–16% of annual deaths were attributed to renal causes compared to ≤ 4% in previous years. Overall, 245 (52%) died from HIV-related causes, 166 (35%) died from non-HIV-related causes, and 63 (13%) died from causes with unknown relatedness to HIV.

### Restricted to participants enrolled from 2013 onwards

Baseline characteristics among the subset of 3956 participants enrolled from 2013 onwards were broadly similar to the overall population enrolled since 2005, except lower proportions of participants had a partner of unknown HIV status (30% among those enrolled in 2013–2018 versus 62% among those enrolled in 2005–2018) and reported alcohol consumption (13% versus 24%, likely due to changes in the way the question was asked, as described in the methods), and higher proportions of participants were referred from in-patient care (16% versus 8%), were diagnosed with tuberculosis (14% versus 6%), were diagnosed with arterial hypertension (13% versus 6%), and initiated ART within 30 days of enrolment (73% versus 52%; Additional file 1: Table S2). Among these participants, 5% of baseline data were missing.

The median follow-up time was 1.4 years (interquartile range 0.6–3.4). Among these participants, 1886 (48%) were in active care at database censoring, 446 (11%) had transferred out to other clinics, 1421 (36%) were LTFU, and 203 (5%) had died. Overall, 64 (32%) deaths were reported to have occurred at home, and 139 (68%) in hospital (of note, place of death was captured from 2013 onwards only). Compared to the overall study population, a larger proportion of deaths occurred in the first six months following enrolment (N = 140, 69%).

**Fig. 2:**
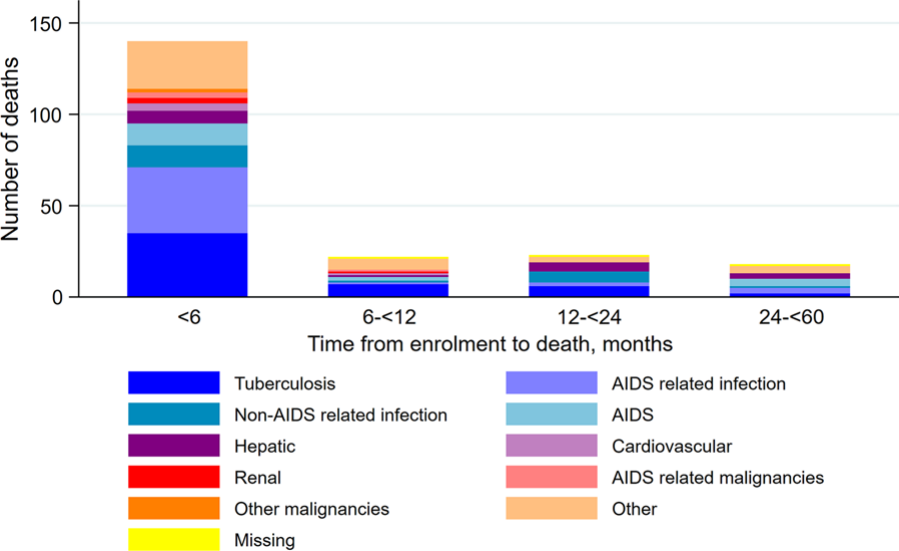
Causes of death among adults enrolled in KIULARCO in 2013–2018, by time since enrolment. The category “AIDS-related infections” excludes tuberculosis which is presented separately

COD was ascertained for 200 participants (99% of those who died). Tuberculosis persisted as the most common COD (25% of all deaths). From six months after enrolment onwards, substantial proportions of deaths were attributable to renal (14%), non-AIDS-related infections (13%), AIDS-related infections (other than tuberculosis; 10%) and cardiovascular (10%) COD, although absolute numbers were small (Fig. 2).

**Fig. 3:**
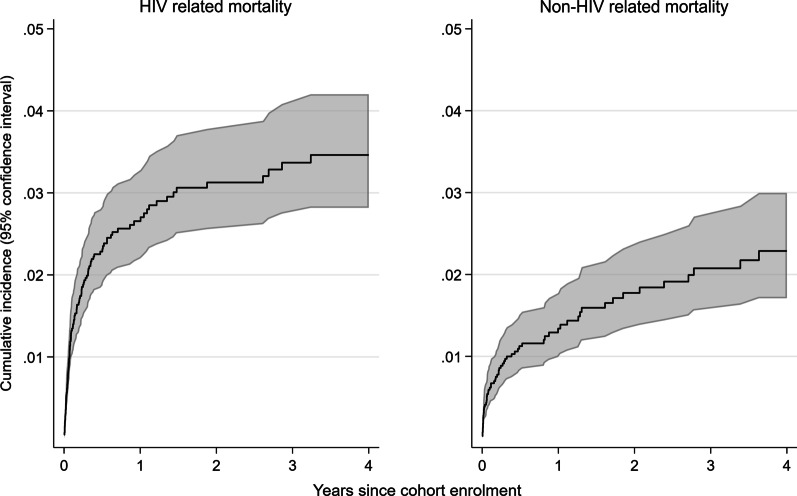
Cause-specific cumulative incidences of HIV-related and non-HIV-related mortality among adults enrolled in KIULARCO in 2013–2018

Overall, 110 (55%) participants died from HIV-related causes, 63 (32%) died from non-HIV-related causes, and 27 (14%) died from causes with unknown relatedness to HIV. HIV-related mortality was especially high within the first few months following enrolment, whereas non-HIV-related mortality increased steadily over time (Fig. 3). The one-year cumulative incidence of HIV-related mortality was 2.7% [95% confidence interval (CI) 2.2–3.2] and of non-HIV-related mortality 1.3% (95% CI 1.0–1.8).

In the multivariable models with multiple imputation for missing covariates, factors associated with higher HIV-related mortality were sex (weak/moderate evidence; adjusted hazard ratio 1.52 (95% confidence interval 1.00–2.31), younger age [for example, 3.10 (1.42–6.75) for 15–24 versus 35–44 years], living in Ifakara town [0.54 (0.33–0.89) for living 2– < 50 km from the clinic compared to in Ifakara town], HIV status disclosure [1.99 (1.12–3.54)], in-patient hospitalisation [2.26 (1.46–3.51)], lower CD4 count [0.29 (0.14–0.61) for ≥ 350 versus < 100 cells/mm^3^], more advanced WHO stage [8.94 (4.23–18.9) for WHO stage IV versus I/II], tuberculosis co-infection [1.93 (1.26–2.95)], and more gaps in care [26.4 (5.61–123) for ≥ 2 versus no previous gaps in care; Table 2]. Being underweight was associated with lower risk of HIV-related mortality [0.56 (0.34–0.94) versus normal BMI]. Factors associated with higher non-HIV-related mortality were not having an HIV-positive partner [3.36 (1.18–9.55) for having an HIV-negative versus HIV-positive partner], lower CD4 count [0.33 (0.16–0.71) for ≥ 350 versus < 100 cells/mm^3^], more advanced WHO stage [2.65 (1.26–5.57) for WHO stage IV versus I/II], and more gaps in care [7.67 (1.49–39.6) for ≥ 2 versus no previous gaps in care]. We also observed lower non-HIV-related mortality among those enrolled in 2013–2014 compared to later years [0.38 (0.17–0.82) for 2013–14 versus 2015–16]. Models on complete cases (i.e., missing baseline data not imputed) yielded similar interpretations (Additional file 1: Table S3).

**Table 2:** Factors associated with HIV- and non-HIV-related mortality among adults enrolled in KIULARCO in 2013–2018

Characteristic	Hazard ratio for HIV-related mortality (95% CI) N = 110 deaths	Hazard ratio for non-HIV-related mortality (95% CI) N = 63 deaths
Sex, male versus female	1.52 (1.00, 2.31)	1.35 (0.77, 2.35)
Age, years		
15–24	3.10 (1.42, 6.75)	0.35 (0.08, 1.61)
25–34	1.31 (0.77, 2.22)	0.96 (0.50, 1.82)
35–44	1 (reference)	1 (reference)
≥ 45	1.60 (0.99, 2.60)	1.17 (0.63, 2.18)
Highest education level, beyond primary versus none/primary	1.20 (0.53, 2.76)	0.54 (0.12, 2.45)
Occupation, not farmer versus farmer	0.61 (0.33, 1.14)	0.65 (0.27, 1.52)
Marital status		
Married/cohabiting	1 (reference)	1 (reference)
Never married	1.14 (0.53, 2.47)	1.57 (0.64, 3.86)
Separated/divorced/widowed/others	1.45 (0.88, 2.40)	0.59 (0.30, 1.19)
Distance of residence from clinic, km		
≤ 1 (i.e., resident in Ifakara town)	1 (reference)	1 (reference)
2– < 50	0.54 (0.33, 0.89)	0.80 (0.44, 1.46)
≥ 50	0.64 (0.39, 1.03)	0.58 (0.29, 1.15)
Partner HIV sero-status		
Positive	1 (reference)	1 (reference)
Negative	2.17 (0.90, 5.22)	3.36 (1.18, 9.55)
Unknown	1.89 (0.89, 4.03)	1.66 (0.59, 4.67)
Not applicable	1.55 (0.71, 3.38)	2.64 (0.95, 7.32)
HIV status disclosed	1.99 (1.12, 3.54)	1.35 (0.68, 2.69)
Smoking, current versus never/stopped	0.76 (0.26, 2.20)	0.31 (0.04, 2.36)
Alcohol use^¥^	1.12 (0.61, 2.06)	0.84 (0.35, 2.02)
Patient referred from in-patient care hospitalization	2.26 (1.46, 3.51)	1.57 (0.84, 2.91)
Pregnant	0.54 (0.07, 4.05)	1.20 (0.27, 5.33)
BMI, kg/m^2^		
Underweight (< 18.5)	0.56 (0.34, 0.94)	0.74 (0.36, 1.51)
Normal (18.5- < 25)	1 (reference)	1 (reference)
Overweight (≥ 25)	0.62 (0.25, 1.54)	0.59 (0.20, 1.70)
CD4 count, cells/mm^3^		
< 100	1 (reference)	1 (reference)
100–349	0.43 (0.27, 0.70)	0.20 (0.10, 0.42)
≥ 350	0.29 (0.14, 0.61)	0.33 (0.16, 0.71)
WHO stage		
I/II	1 (reference)	1 (reference)
III	4.80 (2.29, 10.1)	1.64 (0.82, 3.27)
IV	8.94 (4.23, 18.9)	2.65 (1.26, 5.57)
Tuberculosis	1.93 (1.26, 2.95)	0.88 (0.45, 1.73)
Hypertension	0.92 (0.48, 1.77)	0.98 (0.45, 2.14)
Year of registration		
2013–14	0.59 (0.35, 0.97)	0.38 (0.17, 0.82)
2015–16	1 (reference)	1 (reference)
2017–18	0.76 (0.48, 1.22)	1.47 (0.82, 2.63)
Time since ART initiation during follow-up, months		
Not yet initiated ART	1 (reference)	1 (reference)
0– < 6	0.87 (0.54, 1.41)	1.28 (0.62, 2.66)
≥ 6	0.81 (0.23, 2.90)	1.58 (0.16, 15.2)
Number of previous gaps in care*		
0	1 (reference)	1 (reference)
1	9.51 (3.79, 23.9)	5.23 (2.05, 13.3)
≥ 2	26.4 (5.61, 124)	7.67 (1.49, 39.6)

Results are hazard ratio (95% confidence interval) from multivariable Cox regression model after multiple imputation for missing covariates. ^¥^Captured as ever versus never in 2005–2012, and regular/current versus not regular/current from 2013 onwards. *Periods of being LTFU before returning to care

## Discussion

To our knowledge, this is the first analysis of COD among PLHIV in Tanzania, and covers the period since nationwide ART rollout began in October 2004 through the adoption of universal test-and-treat guidelines in late 2016 [22, 23]. We found a higher incidence of HIV-related deaths than non-HIV-related deaths, even in more recent years, likely due to late presentation and ART initiation at more advanced HIV disease stages [24, 25]. Most deaths occurred within the first six months of enrolment into the cohort, mostly attributable to HIV-related causes. A study in Uganda also observed higher occurrence of HIV-related deaths in the first year on ART, although non-HIV-related COD increased towards the end of ten years of follow-up [8].

Tuberculosis was the leading COD among our study participants despite being only the fifth most prevalent comorbidity in KIULARCO [26]. This is in line with global reports and findings from other SSA countries which identify tuberculosis as a leading cause of death among PLHIV [7, 8, 27, 28]. In our clinic, tuberculosis diagnosis has improved over time by HIV/tuberculosis service integration, systematic adoption of the WHO tuberculosis screening tool, an electronic medical record system, a routine baseline chest radiograph and implementation of GeneXpert [29], and sonography to detect extra-pulmonary manifestations of tuberculosis [30]. However, the challenge of over-diagnosis of tuberculosis remains important, as many patients are diagnosed clinically only without microbiological proof, possibly missing other underlying diseases due to lack of diagnostic tools [31]. Improvement in early tuberculosis case detection, diagnostics, and adequate treatment remains crucial among PLHIV.

We observed the emergence of renal and cardiovascular COD after 2015, contributing to a substantial proportion of deaths occurring from six months after enrolment onwards. This is in concordance with reports of rises in the non-communicable diseases burden among PLHIV in lower- and middle-income countries, including Tanzania [32–34]. The observed changes in COD over time in our cohort may be attributed in part to improved documentation following the introduction of an electronic medical record system, improved screening procedures, and possibly inclusion of hospitalised patients in later years. Notably, studies conducted in KIULARCO in 2015–2016 found prevalences at enrolment of arterial hypertension and renal impairment of 12% and 15%, respectively, with a further 10% and 13% of participants developing these conditions, respectively, during follow-up [35, 36]. These observational studies may have indirectly improved detection and management of such conditions within the cohort. In populations of PLHIV in other settings, a high burden of non-communicable comorbidities such as hypertension, stroke and metabolic disorders has also been demonstrated [33, 37, 38]. In our clinic, we identified anaemia and undernutrition as the most important factors associated with a combined end point of death/lost to follow-up in stable patients on ART besides tuberculosis and opportunistic infections [26]. Early detection and management of non-communicable diseases require more attention within HIV programmes, especially among older people [26].

HIV-related mortality was most predominant in patients with hospitalisation, low CD4 count, advanced WHO stage and tuberculosis co-infection at enrolment. Late presentation of HIV includes patients with opportunistic conditions such as tuberculosis and cryptococcal meningitis, which are associated with high mortality [31, 39]. Besides tuberculosis, which was coded separately, cryptococcosis was the most common HIV-related infection among our study participants and was diagnosed more frequently after implementation of screening with a cryptococcal antigen test, establishment of a referral system for treatment [40], and a trial to evaluate treatment options for cryptococcal meningitis [41]. Being underweight was associated with somewhat lower risk of HIV-related mortality, in contrast to expected [26, 42–44]; the reasons for this are unclear but we note that 15% of participants were missing baseline BMI measurement. Advanced WHO stage and lower CD4 count were also associated with higher risk of non-HIV-related mortality, similar to the findings reported from a 20-year follow-up study among PLHIV in Iran [45]. Chronic HIV has been shown to accelerate and accentuate inflammation and immune dysfunction, which increases the risk of non-HIV-related conditions [46, 47].

Participants who had disclosed their HIV status had a higher risk of HIV-related mortality, which may be attributable in part to hospitalised patients needing to disclose their status. Further, disclosure may perpetuate stigma which is a known cause of poor adherence to treatment and poor outcomes [48]. Having an HIV-positive partner was associated with lower risk of non-HIV-related mortality, and we observed similar trends for HIV-related mortality although the confidence intervals were wide. The support of an HIV-positive partner may foster good adherence to antiretroviral treatment or, conversely, stigma from an HIV-negative partner might impede compliance.

Male gender was associated with somewhat higher risk of HIV-related mortality, as previously observed for overall mortality in this cohort [44]. Younger age was associated with a higher risk of HIV-related mortality, coinciding with evidence that adolescents and young adults have poorer HIV treatment outcomes than older PLHIV [49, 50]. Intermittent periods of LTFU are common in this cohort [51], and gaps in care were strongly associated with a higher risk of mortality from both HIV-related and non-HIV-related COD, indicating that interventions to improve retention in care need to be further strengthened. Living in Ifakara town was associated with a higher risk of HIV-related mortality, perhaps reflecting unreported deaths among those living further from the clinic [44]. Participants enrolled in 2013–14 had a somewhat lower risk of non-HIV-related mortality compared with those enrolled in later years, perhaps attributable to poorer identification of such COD in earlier years and/or residual unmeasured confounding.

This study contributes to the scarce literature on causes of death among PLHIV in a rural sub-Saharan African setting. The study has a number of limitations. Firstly, mortality is likely to have been underestimated due to unseen deaths among participants who were LTFU. In a previous study we found that 40% of participants who were LTFU after enrolment in KIULARCO had died, and accounting for this unseen mortality led to a doubling of five-year mortality estimates [44]. We do not know whether this would apply equally to HIV- and non-HIV-related deaths since it would be challenging to determine COD among those who died after being LTFU. Secondly, we identified COD based on the diagnosis that attending clinicians documented as most likely for participants who died at the St. Francis Referral Hospital, or that community health workers or relatives reported for participants who died elsewhere. The limited diagnostic capacity in this rural setting and lack of forensic or verbal autopsy to ascertain COD may have resulted in inaccurate assignment of COD, especially for participants who died at home. Standardised verbal autopsy procedures have been shown to improve the determination of underlying COD for patients dying outside hospitals [52, 53], but require training of staff and village health workers and are not routinely performed. Thirdly, COD was often not captured before 2013 and improved only after the introduction of an electronic medical record system towards the end of 2012, in line with improvements in data completeness seen in other settings introducing electronic medical records [54]. We therefore restricted the analysis of factors associated with cause-specific deaths to participants enrolled from 2013 onwards only. Fourthly, while we included long-term follow-up covering a decade of clinical care, we do not yet have sufficient data to assess the impacts of universal test-and-treat strategies in reducing HIV-related mortality.

## Conclusions

In conclusion, our study adds important results to the literature on COD among PLHIV in SSA, which can guide screening for important comorbidities among this population. Tuberculosis and HIV-related infections still cause a substantial number of deaths among PLHIV despite improvements in the availability of ART. Timely diagnosis and treatment of HIV and opportunistic infections, and improving retention in care remain essential. Screening of non-communicable comorbidities such as renal and cardiovascular diseases among individuals on ART is important to improve clinical management with the aim of reducing overall mortality among PLHIV. Efforts to improve COD documentation among PLHIV are required to enable progress tracking and shifts in the predominant comorbidities.

## Supplementary Information

**Additional file 1.** Additional Tables S1–S3.

## Data Availability

The datasets generated and analysed during the current study are available in the Zenodo repository, https://doi.org/10.5281/zenodo.5675203.

## Abbreviations

ART: Antiretroviral therapy
BMI: Body mass index
CDCI: Chronic Diseases Clinic of Ifakara
CI: Confidence interval
COD: Cause(s) of death
ICD-10: International cause of death codes, tenth version
KIULARCO: Kilombero and Ulanga Antiretroviral Cohort
LTFU: Lost to follow-up
SSA: Sub-Saharan Africa
