# Supplementary material for: Causes of death and associated factors over a decade of follow-up in a cohort of people living with HIV in rural Tanzania
Source: BMC Infect Dis. 2022 Jan 6;22:37. doi: 10.1186/s12879-021-06962-3 (PMC8739638; doi:10.1186/s12879-021-06962-3)
Supplement: Supplementary file 1 — Additional file 1. Additional Tables S1–S3. [file 12879_2021_6962_MOESM1_ESM.pdf]

ADDITIONAL MATERIAL

Additional Table S1. Causes of death among adults enrolled in KIULARCO.

ICD-10 code	Cause of death	No. deaths	Category	HIV-related
A16	Respiratory tuberculosis, not confirmed bacteriologically or histologically	68	Tuberculosis	Yes
A19.9	Miliary tuberculosis, unspecified	18	Tuberculosis	Yes
A15	Respiratory tuberculosis, bacteriologically and histologically confirmed	13	Tuberculosis	Yes
A16.1	Tuberculosis of lung, bacteriological and histological examination not done	5	Tuberculosis	Yes
A18.3	Tuberculosis of intestines, peritoneum and mesenteric glands	5	Tuberculosis	Yes
A17	Tuberculosis of nervous system	2	Tuberculosis	Yes
A18	Tuberculosis of other organs	2	Tuberculosis	Yes
A19	Miliary tuberculosis	2	Tuberculosis	Yes
A15.0	Tuberculosis of lung, confirmed by sputum microscopy with or without culture	1	Tuberculosis	Yes
A15.6	Tuberculous pleurisy, confirmed bacteriologically and histologically	1	Tuberculosis	Yes
A16.0	Tuberculosis of lung, bacteriologically and histologically negative	1	Tuberculosis	Yes
A16.2	Tuberculosis of lung, without mention of bacteriological or histological confirmation	1	Tuberculosis	Yes
A16.9	Respiratory tuberculosis unspecified, without mention of bacteriological or histological confirmation	1	Tuberculosis	Yes
A17.0	Tuberculous meningitis	1	Tuberculosis	Yes
A17.9	Tuberculosis of nervous system, unspecified	1	Tuberculosis	Yes
A18.0	Tuberculosis of bones and joints	1	Tuberculosis	Yes
A18.1	Tuberculosis of genitourinary system	1	Tuberculosis	Yes
A18.2	Tuberculous peripheral lymphadenopathy	1	Tuberculosis	Yes
A18.7	Tuberculosis of adrenal glands	1	Tuberculosis	Yes
A31.8	Other mycobacterial infections	1	Tuberculosis	Yes
B20.6	HIV disease resulting in Pneumocystis jirovecii pneumonia	21	AIDS-related infections	Yes
B45.1	Cerebral cryptococcosis	21	AIDS-related infections	Yes
B45	Cryptococcosis	8	AIDS-related infections	Yes
B45.7	Disseminated cryptococcosis	3	AIDS-related infections	Yes
B20.7	HIV disease resulting in multiple infections	2	AIDS-related infections	Yes
B58.2	Toxoplasma meningoencephalitis	2	AIDS-related infections	Yes
B20.9	HIV disease resulting in unspecified infectious or parasitic disease	1	AIDS-related infections	Yes

ICD-10 code	Cause of death	No. deaths	Category	HIV-related
B58	Toxoplasmosis	1	AIDS-related infections	Yes
E41	Nutritional marasmus	5	Others	Yes
A68	Relapsing fevers	4	Others	Yes
G03	Meningitis due to other and unspecified causes	4	Others	Yes
A86	Unspecified viral encephalitis	1	Others	Yes
E43	Unspecified severe protein-energy malnutrition	1	Others	Yes
G04.2	Bacterial meningoencephalitis and meningomyelitis, not elsewhere classified	1	Others	Yes
G05	Encephalitis, myelitis and encephalomyelitis in diseases classified elsewhere	1	Others	Yes
G05.0	Encephalitis, myelitis and encephalomyelitis in bacterial diseases classified elsewhere	1	Others	Yes
R64	Cachexia	1	Others	Yes
B20	Human immunodeficiency virus [HIV] disease resulting in infectious and parasitic diseases	13	AIDS	Yes
B22	Human immunodeficiency virus [HIV] disease resulting in other specified diseases	2	AIDS	Yes
B22.0	HIV disease resulting in encephalopathy	2	AIDS	Yes
B22.2	HIV disease resulting in wasting syndrome	2	AIDS	Yes
B22.7	HIV disease resulting in multiple diseases classified elsewhere	2	AIDS	Yes
B23.2	HIV disease resulting in haematological and immunological abnormalities, not elsewhere classified	1	AIDS	Yes
D89.3	Immune reconstitution syndrome	1	AIDS	Yes
C46	Kaposi sarcoma	7	AIDS-related malignancies	Yes
B21.0	HIV disease resulting in Kaposi sarcoma	2	AIDS-related malignancies	Yes
B21.2	HIV disease resulting in other types of non-Hodgkin lymphoma	1	AIDS-related malignancies	Yes
C53	Malignant neoplasm of cervix uteri	1	AIDS-related malignancies	Yes
A41.9	Sepsis, unspecified	2	Non-AIDS-related infections	Yes
A41.8	Other specified sepsis	1	Non-AIDS-related infections	Yes
G00.8	Other bacterial meningitis	1	Non-AIDS-related infections	Yes
G00.9	Bacterial meningitis, unspecified	1	Non-AIDS-related infections	Yes
G01	Meningitis in bacterial diseases classified elsewhere	1	Non-AIDS-related infections	Yes

ICD-10 code	Cause of death	No. deaths	Category	HIV-related
R57.2	Septic shock	4	Others	No
E11	Type 2 diabetes mellitus	2	Others	No
E11.6	Type 2 diabetes mellitus	2	Others	No
J80	Adult respiratory distress syndrome	2	Others	No
R65.2	Systemic Inflammatory Response Syndrome of non-infectious origin without organ failure	2	Others	No
X70	Intentional self-harm by hanging, strangulation and suffocation	2	Others	No
D69.4	Other primary thrombocytopenia	1	Others	No
E53.1	Pyridoxine deficiency	1	Others	No
E86	Volume depletion	1	Others	No
E87.8	Other disorders of electrolyte and fluid balance, not elsewhere classified	1	Others	No
F10.2	Mental and behavioural disorders due to use of alcohol	1	Others	No
G43	Migraine	1	Others	No
G46.8	Other vascular syndromes of brain in cerebrovascular diseases	1	Others	No
G81.9	Hemiplegia, unspecified	1	Others	No
I26.9	Pulmonary embolism without mention of acute cor pulmonale	1	Others	No
J44.9	Chronic obstructive pulmonary disease, unspecified	1	Others	No
J45	Asthma	1	Others	No
J69	Pneumonitis due to solids and liquids	1	Others	No
J69.0	Pneumonitis due to food and vomit	1	Others	No
J96.0	Acute respiratory failure	1	Others	No
K25	Gastric ulcer	1	Others	No
K27	Peptic ulcer, site unspecified	1	Others	No
K27.4	Peptic ulcer, site unspecified	1	Others	No
K35	Acute appendicitis	1	Others	No
K40	Inguinal hernia	1	Others	No
K56.6	Other and unspecified intestinal obstruction	1	Others	No
O03	Spontaneous abortion	1	Others	No
O67.9	Intrapartum haemorrhage, unspecified	1	Others	No
R09.2	Respiratory arrest	1	Others	No
T14.1	Open wound of unspecified body region	1	Others	No
T14.9	Injury, unspecified	1	Others	No
T63.0	Snake venom	1	Others	No
V24	Motorcycle rider injured in collision with heavy transport vehicle or bus	1	Others	No
W74	Unspecified drowning and submersion	1	Others	No
X26	Contact with venomous marine animals and plants	1	Others	No
B50	Plasmodium falciparum malaria	18	Non-AIDS-related infections	No
J18	Pneumonia, organism unspecified	18	Non-AIDS-related infections	No

ICD-10 code	Cause of death	No. deaths	Category	HIV-related
B54	Unspecified malaria	8	Non-AIDS-related infections	No
A09	Other gastroenteritis and colitis of infectious and unspecified origin	7	Non-AIDS-related infections	No
J15	Bacterial pneumonia, not elsewhere classified	4	Non-AIDS-related infections	No
A09.9	Gastroenteritis and colitis of unspecified origin	2	Non-AIDS-related infections	No
A03	Shigellosis	1	Non-AIDS-related infections	No
A05	Other bacterial foodborne intoxications, not elsewhere classified	1	Non-AIDS-related infections	No
A09.0	Other and unspecified gastroenteritis and colitis of infectious origin	1	Non-AIDS-related infections	No
A92.4	Rift Valley fever	1	Non-AIDS-related infections	No
B74.0	Filariasis due to Wuchereria bancrofti	1	Non-AIDS-related infections	No
J18.0	Bronchopneumonia, unspecified	1	Non-AIDS-related infections	No
J18.9	Pneumonia, unspecified	1	Non-AIDS-related infections	No
N39.0	Urinary tract infection, site not specified	1	Non-AIDS-related infections	No
O85	Puerperal sepsis	1	Non-AIDS-related infections	No
I10	Essential (primary) hypertension	5	Cardiovascular	No
I46.9	Cardiac arrest, unspecified	5	Cardiovascular	No
I64	Stroke, not specified as haemorrhage or infarction	5	Cardiovascular	No
I46	Cardiac arrest	3	Cardiovascular	No
I46.1	Sudden cardiac death, so described	3	Cardiovascular	No
I50	Heart failure	3	Cardiovascular	No
I27.9	Pulmonary heart disease, unspecified	2	Cardiovascular	No
R57.1	Hypovolaemic shock	2	Cardiovascular	No
I25.3	Aneurysm of heart	1	Cardiovascular	No
I26	Pulmonary embolism	1	Cardiovascular	No
I95	Hypotension	1	Cardiovascular	No
R57.0	Cardiogenic shock	1	Cardiovascular	No
N17	Acute renal failure	11	Renal	No
N17.9	Acute renal failure, unspecified	1	Renal	No
C25	Malignant neoplasm of pancreas	2	Other malignancies	No
C18.7	Malignant neoplasma of the Sigmoid colon	1	Other malignancies	No
C25.0	Malignant neoplasma of the Head of pancreas	1	Other malignancies	No

ICD-10 code	Cause of death	No. deaths	Category	HIV-related
C25.1	Malignant neoplasma of the Body of pancreas	1	Other malignancies	No
C38.3	Mediastinum, part unspecified	1	Other malignancies	No
C69.0	Malignant neoplasma of the Conjunctiva	1	Other malignancies	No
C76.0	Malignant neoplasma of the Head, face and neck	1	Other malignancies	No
B18	Chronic viral hepatitis	1	Hepatic	No
B18.1	Chronic viral hepatitis B without delta-agent	1	Hepatic	No
K71.2	Toxic liver disease with acute hepatitis	1	Hepatic	No
K72	Hepatic failure, not elsewhere classified	1	Hepatic	No
K76.7	Hepatorenal syndrome	1	Hepatic	No
D64.9	Anaemia, unspecified	18	Others	Unknown
D50	Iron deficiency anaemia	8	Others	Unknown
R50.9	Fever, unspecified	5	Others	Unknown
R50	Fever of other and unknown origin	2	Others	Unknown
R96	Other sudden death, cause unknown	2	Others	Unknown
G09	Sequelae of inflammatory diseases of central nervous system	1	Others	Unknown
G37.9	Demyelinating disease of central nervous system, unspecified	1	Others	Unknown
R07	Pain in throat and chest	1	Others	Unknown
D50.9	Iron deficiency anaemia, unspecified	1	Others	Unknown
G40	Epilepsy	1	Others	Unknown
G93.5	Compression of brain	1	Others	Unknown
R98	Unattended death	1	Others	Unknown
R99	Other ill-defined and unspecified causes of mortality	1	Others	Unknown
I42	Cardiomyopathy	1	Cardiovascular	Unknown
N18	Chronic kidney disease	9	Renal	Unknown
N19	Unspecified kidney failure	5	Renal	Unknown
N18.5	Chronic kidney disease, stage 5	2	Renal	Unknown
N18.3	Chronic kidney disease, stage 3	1	Renal	Unknown
N18.4	Chronic kidney disease, stage 4	1	Renal	Unknown
N18.9	Chronic kidney disease, unspecified	1	Renal	Unknown
-	Missing	452	Unknown	Unknown

Sorted by HIV-relatedness, then category, then from highest to lowest number of deaths within category, then alphabetically by ICD-10 code. AIDS-related infections excludes tuberculosis which is classified separately.

Additional Table S2. Baseline characteristics among adults enrolled in KIULARCO in 2013-2018.

Characteristic	Censored†	Died from non-HIV-related cause	Died from HIV-related cause	Died from unknown cause	Total
Total	3753 (95%)	63 (2%)	110 (3%)	30 (1%)	3956 (100%)
Sex					
Male	1307 (93%)	28 (2%)	57 (4%)	11 (1%)	1403 (100%)
Female	2446 (96%)	35 (1%)	53 (2%)	19 (1%)	2553 (100%)
Age, years					
15-24	362 (96%)	2 (1%)	12 (3%)	2 (1%)	378 (100%)
25-34	1084 (95%)	18 (2%)	27 (2%)	11 (1%)	1140 (100%)
35-44	1277 (96%)	23 (2%)	32 (2%)	3 (<1%)	1335 (100%)
≥45	1030 (93%)	24 (2%)	39 (4%)	14 (1%)	1103 (100%)
Highest education level					
None/ primary	3461 (95%)	61 (2%)	102 (3%)	30 (1%)	3654 (100%)
Beyond primary	292 (97%)	2 (1%)	8 (3%)	0 (0%)	302 (100%)
Occupation					
Farmer	3120 (95%)	56 (2%)	94 (3%)	25 (1%)	3295 (100%)
Not farmer	633 (96%)	7 (1%)	16 (2%)	5 (1%)	661 (100%)
Marital status					
Married/ cohabiting	2274 (95%)	39 (2%)	57 (2%)	15 (1%)	2385 (100%)
Never married	349 (94%)	8 (2%)	12 (3%)	4 (1%)	373 (100%)
Separated/ divorced/ widowed/ others	1130 (94%)	16 (1%)	41 (3%)	11 (1%)	1198 (100%)
Distance of residence from clinic, km					
≤1 (i.e. resident in Ifakara town)	1389 (94%)	29 (2%)	49 (3%)	13 (1%)	1480 (100%)
2-<50	1205 (96%)	20 (2%)	26 (2%)	8 (1%)	1259 (100%)
≥50	994 (95%)	12 (1%)	31 (3%)	9 (1%)	1046 (100%)
Missing	165 (96%)	2 (1%)	4 (2%)	0 (0%)	171 (100%)
Partner HIV sero-status					
Positive	685 (98%)	5 (1%)	9 (1%)	3 (<1%)	702 (100%)
Negative	372 (93%)	11 (3%)	12 (3%)	3 (1%)	398 (100%)
Unknown	1008 (95%)	15 (1%)	36 (3%)	6 (1%)	1065 (100%)
Not applicable	1270 (93%)	27 (2%)	49 (4%)	16 (1%)	1362 (100%)
Missing	418 (97%)	5 (1%)	4 (1%)	2 (<1%)	429 (100%)
HIV status disclosure					
Not disclosed	774 (96%)	11 (1%)	14 (2%)	7 (1%)	806 (100%)
Disclosed	2561 (94%)	47 (2%)	92 (3%)	21 (1%)	2721 (100%)
Missing	418 (97%)	5 (1%)	4 (1%)	2 (<1%)	429 (100%)
Smoking					
Never/ stopped	3205 (95%)	57 (2%)	102 (3%)	26 (1%)	3390 (100%)
Current	137 (95%)	1 (1%)	4 (3%)	2 (1%)	144 (100%)

Characteristic	Censored†	Died from non-HIV-related cause	Died from HIV-related cause	Died from unknown cause	Total
Missing	411 (97%)	5 (1%)	4 (1%)	2 (<1%)	422 (100%)
Alcohol use[¥]					
No	2944 (94%)	55 (2%)	95 (3%)	26 (1%)	3120 (100%)
Yes	461 (95%)	6 (1%)	14 (3%)	4 (1%)	485 (100%)
Missing	348 (99%)	2 (1%)	1 (<1%)	0 (0%)	351 (100%)
Patient referred from in-patient care hospitalisation					
No	3172 (96%)	45 (1%)	66 (2%)	26 (1%)	3309 (100%)
Yes	581 (90%)	18 (3%)	44 (7%)	4 (1%)	647 (100%)
Pregnant*					
No	2238 (96%)	33 (1%)	52 (2%)	17 (1%)	2340 (100%)
Yes	208 (98%)	2 (1%)	1 (<1%)	2 (1%)	213 (100%)
BMI, kg/m^2‡					
Underweight (<18.5)	588 (93%)	12 (2%)	23 (4%)	8 (1%)	631 (100%)
Normal (18.5-<25)	2052 (94%)	39 (2%)	73 (3%)	16 (1%)	2180 (100%)
Overweight (≥25)	587 (98%)	4 (1%)	6 (1%)	4 (1%)	601 (100%)
Missing	318 (96%)	6 (2%)	7 (2%)	0 (0%)	331 (100%)
CD4 count, cells/mm^3					
<100	710 (88%)	35 (4%)	55 (7%)	11 (1%)	811 (100%)
100-359	1245 (97%)	9 (1%)	26 (2%)	10 (1%)	1290 (100%)
≥350	1049 (97%)	11 (1%)	10 (1%)	6 (1%)	1076 (100%)
Missing	749 (96%)	8 (1%)	19 (2%)	3 (<1%)	779 (100%)
WHO stage					
I/II	1930 (98%)	18 (1%)	10 (1%)	13 (1%)	1971 (100%)
III	989 (93%)	22 (2%)	44 (4%)	9 (1%)	1064 (100%)
IV	460 (85%)	21 (4%)	54 (10%)	7 (1%)	542 (100%)
Missing	374 (99%)	2 (1%)	2 (1%)	1 (<1%)	379 (100%)
Tuberculosis					
Unlikely	3160 (96%)	48 (1%)	58 (2%)	23 (1%)	3289 (100%)
Yes	474 (88%)	12 (2%)	44 (8%)	6 (1%)	536 (100%)
Missing	119 (91%)	3 (2%)	8 (6%)	1 (1%)	131 (100%)
Hypertension					
No	3259 (95%)	55 (2%)	99 (3%)	22 (1%)	3435 (100%)
Yes	494 (95%)	8 (2%)	11 (2%)	8 (2%)	521 (100%)
ART status (within 30 days of enrolment)					
Not yet initiated ART	983 (93%)	17 (2%)	48 (5%)	8 (1%)	1056 (100%)
Initiated ART	2770 (96%)	46 (2%)	62 (2%)	22 (1%)	2900 (100%)

Results are number and row percentage. †Due to administrative censoring at time of database closure, LTFU or transfer out to another clinic (see methods). [¥]Captured as ever versus never in 2005-2012, and regular/current versus not regular/current from 2013 onwards. *Percentages are of females. ‡Pregnant women excluded.

Additional Table S3. Factors associated with HIV- and non-HIV-related mortality among adults enrolled in KIULARCO in 2013-2018 (complete cases).

Characteristic	HIV-related mortality (N=110 deaths)		Non-HIV-related mortality (N=63 deaths)	
	**Univariable**	**Multivariable***	**Univariable**	**Multivariable†**
Sex, male versus female	2.04 (1.40,2.97)	1.45 (0.88,2.40)	1.56 (0.95,2.57)	1.68 (0.87,3.26)
Age, years				
15-24	1.37 (0.71,2.67)	4.26 (1.71,10.6)	0.33 (0.08,1.41)	0.71 (0.14,3.70)
25-34	0.99 (0.59,1.66)	1.04 (0.54,1.99)	0.94 (0.50,1.73)	1.06 (0.47,2.41)
35-44	1 (reference)	1 (reference)	1 (reference)	1 (reference)
≥45	1.50 (0.94,2.39)	1.34 (0.76,2.36)	1.07 (0.59,1.95)	1.65 (0.79,3.46)
Highest education level, beyond primary versus none/ primary	0.94 (0.46,1.93)	1.22 (0.43,3.51)	0.40 (0.10,1.62)	0.78 (0.16,3.75)
Occupation, not farmer versus farmer	0.84 (0.49,1.42)	0.63 (0.28,1.39)	0.62 (0.28,1.36)	0.68 (0.26,1.82)
Marital status				
Married/ cohabiting	1 (reference)	1 (reference)	1 (reference)	1 (reference)
Never married	1.40 (0.75,2.61)	0.86 (0.32,2.36)	1.41 (0.66,3.01)	1.33 (0.42,4.16)
Separated/ divorced/ widowed/ others	1.44 (0.96,2.15)	1.54 (0.84,2.81)	0.82 (0.46,1.46)	0.45 (0.19,1.08)
Distance of residence from clinic, km				
≤1 (i.e. resident in Ifakara town)	1 (reference)	1 (reference)	1 (reference)	1 (reference)
2-<50	0.64 (0.40,1.04)	0.72 (0.41,1.25)	0.86 (0.48,1.52)	0.80 (0.41,1.56)
≥50	0.99 (0.63,1.55)	0.63 (0.35,1.12)	0.67 (0.34,1.32)	0.47 (0.20,1.08)
Partner HIV sero-status				
Positive	1 (reference)	1 (reference)	1 (reference)	1 (reference)
Negative	2.47 (1.04,5.87)	1.93 (0.69,5.36)	4.12 (1.43,11.9)	4.58 (1.41,14.8)
Unknown	2.81 (1.35,5.83)	1.62 (0.68,3.84)	2.11 (0.77,5.80)	1.97 (0.61,6.32)
Not applicable	3.00 (1.47,6.10)	1.43 (0.57,3.57)	3.00 (1.16,7.80)	3.20 (0.96,10.7)
HIV status disclosed, versus not disclosed	1.90 (1.08,3.34)	1.96 (0.99,3.88)	1.23 (0.64,2.37)	1.60 (0.70,3.68)
Smoking, current versus never/stopped	0.91 (0.34,2.48)	0.98 (0.33,2.94)	0.41 (0.06,2.99)	(omitted)
Alcohol use[¥]	0.93 (0.53,1.64)	0.96 (0.47,1.97)	0.69 (0.30,1.60)	0.53 (0.18,1.53)
Patient referred from in-patient care hospitalization	4.32 (2.94,6.34)	1.81 (1.05,3.13)	2.83 (1.63,4.91)	1.98 (0.96,4.09)
Pregnant	0.16 (0.02,1.12)	(omitted)	0.55 (0.13,2.25)	(omitted)
BMI, kg/m^2				
Underweight (<18.5)	1.11 (0.69,1.77)	0.52 (0.29,0.92)	1.06 (0.55,2.02)	0.62 (0.28,1.38)
Normal (18.5-<25)	1 (reference)	1 (reference)	1 (reference)	1 (reference)
Overweight (≥25)	0.29 (0.12,0.66)	0.38 (0.12,1.28)	0.35 (0.13,0.98)	0.65 (0.22,1.90)
CD4 count, $cells/mm^3$				
<100	1 (reference)	1 (reference)	1 (reference)	1 (reference)

Characteristic	HIV-related mortality (N=110 deaths)		Non-HIV-related mortality (N=63 deaths)	
	**Univariable**	**Multivariable***	**Univariable**	**Multivariable†**
100-349	0.28 (0.17,0.44)	0.40 (0.23,0.68)	0.15 (0.07,0.31)	0.18 (0.08,0.40)
≥350	0.13 (0.07,0.25)	0.26 (0.11,0.58)	0.23 (0.11,0.45)	0.31 (0.13,0.71)
WHO stage				
I/II	1 (reference)	1 (reference)	1 (reference)	1 (reference)
III	8.59 (4.32,17.1)	4.64 (2.03,10.6)	2.41 (1.29,4.49)	1.37 (0.64,2.95)
IV	23.0 (11.7,45.2)	8.32 (3.53,19.6)	5.11 (2.72,9.60)	1.66 (0.68,4.04)
Tuberculosis	4.99 (3.37,7.38)	1.70 (1.04,2.76)	1.61 (0.86,3.04)	0.82 (0.38,1.77)
Hypertension	0.68 (0.36,1.27)	1.23 (0.60,2.49)	0.86 (0.41,1.81)	0.99 (0.42,2.34)
Year of registration				
2013-14	0.49 (0.30,0.80)	0.60 (0.33,1.12)	0.37 (0.17,0.79)	0.34 (0.12,0.92)
2015-16	1 (reference)	1 (reference)	1 (reference)	1 (reference)
2017-18	0.65 (0.41,1.02)	0.90 (0.51,1.60)	1.43 (0.82,2.51)	1.43 (0.71,2.85)
Time since ART initiation during follow-up, months				
Not yet initiated ART	1 (reference)	1 (reference)	1 (reference)	1 (reference)
0-<6	0.73 (0.47,1.13)	0.49 (0.28,0.89)	1.26 (0.63,2.52)	0.72 (0.30,1.75)
≥6	0.57 (0.18,1.82)	0.43 (0.11,1.69)	1.79 (0.21,15.4)	0.68 (0.06,7.32)
Number of previous gaps in care#				
0	1 (reference)	1 (reference)	1 (reference)	1 (reference)
1	8.47 (3.48,20.6)	12.7 (4.65,34.9)	4.71 (1.89,11.8)	3.66 (1.03,13.1)
≥2	25.1 (5.56,113)	28.8 (2.62,316)	5.90 (1.16,30.1)	13.4 (2.33,76.8)

Results are hazard ratio (95% confidence interval) from Cox regression models on participants with non-missing covariates. Pregnancy omitted from the multivariable models due to the inclusion of BMI in the model, which is always missing for pregnant women. *Model fitted based on 75 deaths in 2508 participants. †In the subset of participants with complete baseline data, there were no non-HIV-related deaths among current smokers, therefore this variable was omitted from the model. Model fitted based on 47 deaths in 2508 participants. ¥Captured as ever versus never in 2005-2012, and regular/current versus not regular/current from 2013 onwards. #Periods of being LTFU before returning to care.
